# Impact of EGFR variant allele frequency on treatment-related adverse events in patients with metastatic NSCLC treated with osimertinib

**DOI:** 10.3389/fmolb.2026.1878152

**Published:** 2026-06-24

**Authors:** Walid Shalata, Bilal Krayim, Asmah Miari, Abed Agbarya, Irina Lazarev, Nir Peled, Yulia Dudnik, Ahron Yehonatan Cohen, Amichay Meirovitz, Natalie Maimon Rabinovich, Alexander Yakobson, Firas Abu Akar, Ronen Brenner

**Affiliations:** 1 The Legacy Heritage Cancer Center and Dr. Larry Norton Institute, Soroka Medical Center, Be'er Sheva, Israel; 2 Ben-Gurion University of the Negev, Be'er Sheva, Israel; 3 Helmsley Cancer Center, Shaare Zedek Medical Center, Jerusalem, Israel; 4 Department of Oncology, Meir Medical Center, Kfar Saba, Israel; 5 Oncology Department, Bnai Zion Medical Center, Haifa, Israel; 6 Department of Oncology, Assuta Ashdod Medical Center, Ashdod, Israel; 7 Edith Wolfson Medical Center, Oncology Institute, Holon, Israel; 8 Faculty of Medicine, Tel Aviv University, Tel Aviv, Israel

**Keywords:** epidermal growth factor receptor, non-small cell lung cancer, osimertinib, overall survival, progression-free survival, treatment-related adverse events, variant allele frequency

## Abstract

**Background:**

Osimertinib is an approved first-line therapy for epidermal growth factor receptor (EGFR)-mutated non-small cell lung cancer (NSCLC). However, beyond the identification of common EGFR mutations, additional pathological or molecular factors that predict treatment response, survival outcomes, or toxicity remain limited.

**Materials and Methods:**

This retrospective study analyzed data from a registry of NSCLC patients with EGFR mutations treated with first-line osimertinib between March 2017 and December 2024. Variant allele frequency (VAF) was evaluated as a potential predictive factor for overall survival (OS), progression-free survival (PFS), and adverse events (AEs).

**Results:**

Among 147 eligible patients, the mean OS was 25.5 months and the mean PFS was 21.4 months. Patients with VAF ≥30% exhibited improved outcomes compared to those with VAF <30%, with mean OS of 31.4 months versus 19.7 months (p = 0.022), and mean PFS of 25.0 months versus 18.2 months (p = 0.234). Similar trends were observed across EGFR exon 19 deletion and exon 21 L858R subgroups (p = 0.056). When comparing toxicity profiles, the overall AE rates were similar between high-VAF and low-VAF patients. However, several statistically significant differences were noted: diarrhea (21.3% vs. 5.7%, p = 0.005) and dyspnea (16.4% vs. 3.4%, p = 0.0085) were more frequent in the high-VAF group, while anemia (9.2% vs. 3.3%, p = 0.03) and creatinine elevation (5.7% vs. 1.6%, p = 0.01) occurred more commonly in the low-VAF group.

**Conclusion:**

Higher EGFR VAF was significantly associated with improved overall survival in patients with EGFR-mutated NSCLC treated with first-line osimertinib and showed a numerical trend toward longer progression-free survival. Similar patterns were observed across key molecular subgroups. Additionally, this study is the first to report potential VAF-related differences in adverse event patterns, suggesting that VAF may have relevance not only for efficacy but also for toxicity characterization. These findings support the potential role of VAF as a prognostic biomarker in EGFR-mutant NSCLC and warrant further prospective validation.

## 1 Introduction

Lung cancer continues to be the most common cause of cancer-related death globally and ranks as the second most frequently diagnosed malignancy. Current epidemiological data suggest that it represents roughly 12% of all newly diagnosed cancers, following prostate cancer in men (≈26%) and breast cancer in women (≈30%). Despite its relatively lower incidence compared with these malignancies, lung cancer accounts for the greatest cancer-related mortality, responsible for nearly 45% of all cancer deaths in both sexes. The vast majority of cases are classified as non-small cell lung cancer (NSCLC), which includes adenocarcinoma and squamous cell carcinoma, and constitutes approximately 85% of all lung cancer diagnoses (Sung et al., 2021; Sh et al., 2024; Shalata et al., 2021).

Activating somatic mutations in the tyrosine kinase domain of the epidermal growth factor receptor (EGFR) are present in approximately 15% of White patients and up to 50% of Asian patients with advanced NSCLC. EGFR tyrosine kinase inhibitors (TKIs) act by competitively inhibiting ATP binding within the EGFR kinase domain, thereby blocking receptor autophosphorylation and downstream signalling pathways essential for tumor growth and survival. Through this mechanism, EGFR-TKIs effectively reduce tumor proliferation and metastatic potential, and have become a foundational therapeutic strategy in EGFR-mutated NSCLC. Their use has led to substantial improvements in progression-free survival (PFS) and objective response rates (ORR), with more modest gains in overall survival (OS) (Zhang et al., 2016; Tan et al., 2015; Tan et al., 2018; Cross et al., 2014).

Currently, three generations of EGFR-TKIs are available in clinical practice. First-generation agents, including gefitinib, erlotinib, and icotinib, and second-generation agents such as afatinib and dacomitinib, have shown robust activity in tumors harbouring classical sensitizing mutations, particularly exon 19 deletions and exon 21 L858R substitutions (Soria et al., 2018; Lamb, 2021).

Osimertinib, a third-generation, irreversible EGFR-TKI, was designed to selectively inhibit both sensitizing mutations and the T790M resistance mutation. It achieves this through covalent binding to cysteine 797 within the ATP-binding pocket of the EGFR kinase domain. The pivotal FLAURA trial, a randomized phase III study comparing osimertinib with first-generation EGFR-TKIs, demonstrated superior efficacy and established osimertinib as the preferred first-line treatment for metastatic EGFR-mutated NSCLC (Soria et al., 2018; Lamb, 2021; Meng et al., 2023; D'Angelo et al., 2011).

Variant allele frequency (VAF) reflects the proportion of sequencing reads carrying a specific mutation at a given genomic locus and may provide insight into tumor clonal architecture and heterogeneity. It can also assist in differentiating driver from passenger mutations and identifying potential germline alterations. Although computational reconstruction of clonal evolution remains complex, VAF is a readily obtainable biomarker in clinical practice (Friedlaender et al., 2021; Gieszer et al., 2021).

Recent evidence suggests that variant allele frequency (VAF) is a significant predictor of overall survival (OS) and progression-free survival (PFS) in patients with EGFR-mutant NSCLC treated with osimertinib (Shalata et al., 2026). However, its impact on treatment-related adverse events (TRAEs) remains insufficiently characterized. Therefore, this study aims to evaluate the association between baseline EGFR VAF and clinical outcomes, with a particular focus on its influence on TRAEs, in patients with metastatic EGFR-mutant NSCLC receiving osimertinib in a real-world setting.

## 2 Materials and methods

### 2.1 Study design and population

This was a retrospective, single-cohort observational study including patients with advanced non-small cell lung cancer (NSCLC) harboring activating *EGFR* mutations. Eligible patients were treated with first-line osimertinib between March 2017 and December 2024, with follow-up data collected through December 2024. The primary objective was to evaluate the association between *EGFR* mutation subtype, VAF, and clinical outcomes, including OS, PFS, and TRAEs.

Clinical data were extracted from institutional medical records and included demographic characteristics, smoking history, metastatic burden, *EGFR* mutation subtype (exon 19 deletion vs. exon 21 (L858R), ECOG performance status, treatment timelines, sites of progression, survival outcomes, and adverse events.

An exploratory minimal p-value approach was performed to evaluate the association between VAF and overall survival across multiple candidate cut-off values. For each threshold, patients were dichotomized into high- and low-VAF groups and compared using the log-rank test. The threshold yielding the smallest p-value was considered the optimal cut-off. This analysis was performed as a *post hoc* exploratory assessment to evaluate the robustness of the pre-specified 30% threshold used in the primary analyses.

### 2.2 Multidisciplinary evaluation and treatment strategy

All patients were managed within a multidisciplinary tumor board setting involving medical oncology, pulmonology, radiation oncology, pathology, radiology, nuclear medicine, and thoracic surgery specialists. Treatment decisions were individualized based on histopathological confirmation, radiological findings, molecular profiling, and patient performance status. For patients with advanced or metastatic disease, systemic therapy decisions followed National Comprehensive Cancer Network (NCCN) guideline recommendations (National Comprehensive Cancer Network et al., 2025).

### 2.3 Baseline assessment and follow-up

Prior to initiation of therapy, all patients underwent comprehensive staging, including brain magnetic resonance imaging (MRI) and systemic imaging with contrast-enhanced computed tomography (CT) and/or fluorodeoxyglucose positron emission tomography-computed tomography (FDG PET-CT).

Tumor response was assessed using Response Evaluation Criteria in Solid Tumors (RECIST). Radiological reassessments were routinely performed every 3–4 months using CT, brain MRI, or FDG PET-CT, depending on disease distribution.

Safety monitoring was conducted throughout treatment using the Common Terminology Criteria for Adverse Events (CTCAE), version 5.0, with systematic documentation of all treatment-emergent toxicities.

### 2.4 Eligibility criteria

#### 2.4.1 Inclusion criteria

Patients were included if they met all of the following criteria:

- Age ≥18 years
- Histologically confirmed NSCLC
- Presence of activating *EGFR* mutation (exon 19 deletion or exon 21 L858R) detected by tissue biopsy
- Availability of baseline variant allele frequency (VAF) data
- Receipt of osimertinib as first-line monotherapy
- Documented follow-up data, including treatment-related adverse events

#### 2.4.2 Exclusion criteria

Patients were excluded if they met any of the following:

- Prior systemic anticancer therapy within 4 years before study entry
- Missing, incomplete, or non-evaluable TRAE data
- Missing, incomplete, or non-evaluable VAF data
- Use of osimertinib beyond the first-line setting
- Absence of documented safety or adverse event information

### 2.5 Endpoints and data handling

The primary endpoints was to verify the TRAEs, OS and PFS. OS was defined as the time from treatment initiation to death from any cause, while PFS was defined as the time from treatment initiation to radiological or clinical disease progression or death, whichever occurred first.

Adverse events were categorized by severity and frequency and analyzed according to mutation subtype and VAF stratification. All data were anonymized prior to analysis to ensure patient confidentiality.

## 3 Results

In this multi-center retrospective real-world cohort of 147 patients with locally advanced or metastatic EGFR-mutant NSCLC treated with osimertinib, the population was predominantly female (74.8%), with a mean age of 71.3 years, and most patients presented with stage IV disease (85.7%) and good performance status (ECOG 0–1 in 82.3%). EGFR exon 19 deletions were slightly more common than exon 21 L858R mutations (54.4% vs. 45.6%), with comparable mean VAF levels between subtypes (36.65% vs. 33.9%). Lung metastases were the most frequent at diagnosis, followed by pleural, bone, and brain involvement (Table 1).

**TABLE 1:** Study population characteristics.

Characteristics		Mean (range)
Age (years)		71.3 (25-90)
	Female	71.1 (38-90)
	Male	71.8 (25-90)
Gender		Frequencies (percentage)
	Female	110 (74.83)
	Male	37 (25.17)
ECOG status (at diagnosis)		
	0	52 (35.37)
	1	69 (46.94)
	2	18 (12.24)
	3	8 (5.44)
Stage at diagnosis		
	3	21 (14.29)
	4	126 (85.71)
Smoking status (at diagnosis)		
	Former or current	40 (27.21)
	Never	107 (72.79)
Histology	Adenocarcinoma	147 (100)
Type of histology	Tissue	147 (100)
EGFR mutation types		Mean (median)
	EGFR ex 19 deletions	80 (54.42)
		Mean (range)
	VAF	36.65 (2.2-96.16)
		Mean (median)
	EGFR ex 21 L858R	67 (45.58)
		Mean (range)
	VAF	33.9 (2.7-94.54)
VAF values		
	≥30	68 (46.2)
	<30	79 (53.8)
Metastasis sites at diagnosis		
Lung		
	Yes	121 (82.31)
	No	26 (17.69)
Pleural		
	Yes	54 (36.73)
	No	93 (63.27)
Bone		
	Yes	52 (35.37)
	No	95 (64.63)
Brain		
	Yes	45 (30.61)
	No	102 (69.39)
Liver		
	Yes	23 (15.65)
	No	124 (84.35)
Adrenal		
	Yes	8 (5.44)
	No	139 (94.56)
Lymph nodes		
	Yes	73 (49.66)
	No	74 (50.34)

Abbreviations: VAF, variant allele frequency.

**FIGURE 1:**
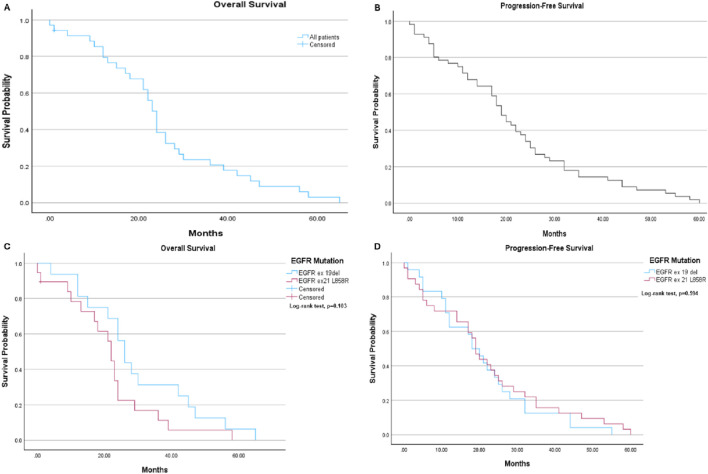
Kaplan–Meier survival curves. **(A)** Mean overall survival (OS) for the entire cohort was 25.5 months (95% CI, 20.28–30.75). **(B)** Mean progression-free survival (PFS) was 21.4 months (95% CI, 17.38–25.37). **(C)** When stratified by EGFR mutation subtype, patients with exon 19 deletion (ex19del) had a longer mean OS of 29.8 months (95% CI, 21.52–38.11) compared with 21.8 months (95% CI, 15.48–28.02) for exon 21 L858R. **(D)** Mean PFS was similar between subgroups, at 20.7 months (95% CI, 15.26–26.16) for ex19del and 21.9 months (95% CI, 16.13–27.62) for L858R.

In the overall cohort, mean overall survival (OS) was 25.5 months (95% CI: 20.28–30.75), (Figure 1A), and progression-free survival (PFS) was 21.4 months (95% CI: 17.38–25.37), (Figure 1B). Patients with exon 19 deletions demonstrated numerically longer OS compared to those with L858R mutations (29.8 months [95% CI: 21.52–38.11] vs. 21.8 months [95% CI: 15.48–28.02]; p = 0.103) (Figure 1C), while PFS was similar between groups (20.7 months [95% CI: 15.26–26.16] vs. 21.9 months [95% CI: 16.13–27.62]; p = 0.594) (Figure 1D).

**FIGURE 2:**
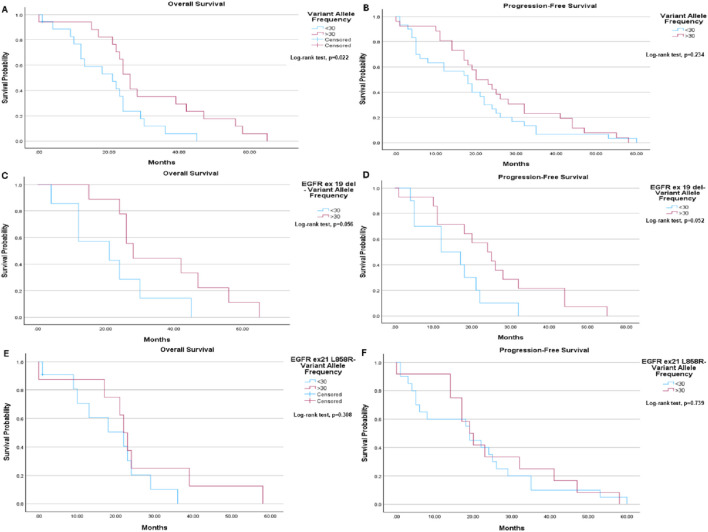
Kaplan–Meier survival curves stratified by variant allele frequency (VAF). **(A)** Mean OS was 19.7 months (95% CI, 14.21–25.10) for VAF <30% and 31.4 months (95% CI, 23.15–39.56) for VAF ≥30%. **(B)** Mean PFS was 18.2 months (95% CI, 12.94–23.46) for VAF <30% and 25.0 months (95% CI, 19.14–30.94) for VAF ≥30%. **(C)** In the EGFR exon 19 deletion subgroup, mean OS was 21.1 months (95% CI, 11.04–31.25) for VAF <30% and 36.6 months (95% CI, 25.61–47.50) for VAF ≥30%. **(D)** Mean PFS in this subgroup was 14.8 months (95% CI, 9.24–20.37) for VAF <30% and 24.9 months (95% CI, 17.04–32.82) for VAF ≥30%. **(E)** Among patients with EGFR exon 21 L858R, mean OS was 18.7 months (95% CI, 12.36–25.00) for VAF <30% and 25.5 months (95% CI, 13.78–37.22) for VAF ≥30%. **(F)** Corresponding mean PFS values were 19.9 months (95% CI, 12.54–27.26) and 25.2 months (95% CI, 15.92–34.41), respectively.

Stratification by VAF revealed that patients with VAF ≥30% had significantly longer OS compared to those with VAF <30% (31.4 months [95% CI: 23.15–39.56] vs. 19.7 months [95% CI: 14.21–25.10]; p = 0.022) (Figure 2A), with a corresponding, though not statistically significant, improvement in PFS (25.0 months [95% CI: 19.14–30.94] vs. 18.2 months [95% CI: 12.94–23.46]; p = 0.234), (Figure 2B). Within the exon 19 deletion subgroup, higher VAF (≥30%) was associated with a trend toward improved OS (36.6 vs. 21.1 months; p = 0.056) (Figure 2C), and PFS (24.9 vs. 14.8 months; p = 0.052) (Figure 2D). In the L858R subgroup, numerical differences in OS (25.5 vs. 18.7 months; p = 0.308) (Figure 2E), and PFS (25.2 vs. 19.9 months; p = 0.739) were observed but were not statistically significant (Figure 2F).

Exploratory analyses showed a non-significant trend toward longer survival in males compared to females (OS: 31.8 months [95% CI: 21.18–42.46] vs. 22.5 months [95% CI: 16.93–28.11]; p = 0.109; PFS: 25.0 months [95% CI: 18.39–31.60] vs. 19.8 months [95% CI: 14.86–24.73]; p = 0.423). Smoking status did not significantly influence outcomes, with similar OS (25.9 months [95% CI: 13.58–38.22] vs. 25.4 months [95% CI: 20.28–30.75]; p = 0.731) and PFS (19.7 months [95% CI: 9.81–29.57] vs. 21.4 months [95% CI: 17.38–25.37]; p = 0.738) observed between smokers and non-smokers. Overall, higher baseline VAF was associated with improved survival outcomes, while differences across other clinical subgroups were not statistically significant.

Patients were stratified according to baseline variant allele frequency (VAF) into low-VAF (<30%) and high-VAF (≥30%) groups. The median duration of osimertinib exposure was approximately 19 months in the low-VAF group and 25 months in the high-VAF group. The corresponding median follow-up durations were approximately 24 months and 34 months, respectively. Patients who remained on treatment or alive at the time of data cutoff (31 December 2024) were censored at that date.

TRAEs were predominantly mild in severity. Fatigue was the most frequently observed AE, occurring in 22.4% of patients, largely as grade 1, with only one grade 3 event. Diarrhea was reported in 18.4% of patients, mainly grade 1–2, with a single grade 3 case. Rash occurred in 17% of patients and was generally low grade. Paronychia and cough were each observed in 7.5% of patients and were mostly mild, aside from one grade 3 paronychia event. Hematologic toxicities, including anemia, alopecia, and thrombocytopenia (each 5.4%), were uncommon, although isolated grade 4 anemia and thrombocytopenia were noted. Other AEs such as dyspnea, chest pain, renal impairment, creatinine elevation, and abdominal pain were infrequent (3.4% each) and limited to grade 1 severity. Additional less common toxicities (1%–3%) included decreased ejection fraction, pancytopenia, decreased appetite, vomiting, headache, pneumonitis, hypercalcemia, elevated transaminases, peripheral neuropathy, and arthralgia. Rare events (0.7%) encompassed a wide range of mostly mild symptoms. Serious but uncommon events included single cases of atrial fibrillation, deep vein thrombosis, and grade 4 pneumonitis. Overall, high-grade (grade 3–4) toxicities were rare, indicating a favorable safety profile (Table 2).

**TABLE 2:** Study population treatment-related adverse events.

Adverse event	Grade 1 (frequency, %)	Grade 2 (frequency, %)	Grade 3 (frequency, %)	Grade 4 (frequency, %)	Total (frequency, %)
Fatigue	32 (21.8%)	0 (0%)	1 (0.7%)	0 (0%)	33 (22.4%)
Diarrhea	13 (8.8%)	13 (8.8%)	1 (0.7%)	0 (0%)	27 (18.4%)
Rash	13 (8.8%)	11 (7.5%)	1 (0.7%)	0 (0%)	25 (17.0%)
Paronychia	6 (4.1%)	4 (2.7%)	1 (0.7%)	0 (0%)	11 (7.5%)
Cough	11 (7.5%)	0	0	0	11 (7.5%)
Mild anemia	4 (2.7%)	3 (2.0%)	0	1 (0.7%)	8 (5.4%)
Alopecia	1 (0.7%)	7 (4.8%)	0	0	8 (5.4%)
Thrombocytopenia	2 (1.4%)	5 (3.4%)	0	1 (0.7%)	8 (5.4%)
Dyspnea	6 (4.1%)	0	0	0	6 (4.1%)
Chest pain	5 (3.4%)	0	0	0	5 (3.4%)
Mild renal failure	5 (3.4%)	0	0	0	5 (3.4%)
Elevated creatinine	5 (3.4%)	0	0	0	5 (3.4%)
Abdominal pain	5 (3.4%)	0	0	0	5 (3.4%)
Decreasing heart ejection fraction	4 (2.8%)	0	0	0	4 (2.8%)
Pancytopenia	2 (1.4%)	0	1 (0.7%)	0	3 (2.0%)
Decreased appetite	3 (2.0%)	0	0	0	3 (2.0%)
Vomiting	2 (1.4%)	0	0	0	2 (1.4%)
Headaches	1 (0.7%)	1 (0.7%)	0	0	2 (1.4%)
Pneumonitis	1 (0.7%)	0	0	1 (0.7%)	2 (1.4%)
Hypercalcemia	2 (1.4%)	0	0	0	2 (1.4%)
Transaminases	2 (1.4%)	0	0	0	2 (1.4%)
Peripheral neuropathy	0	2 (1.4%)	0	0	2 (1.4%)
Arthralgia	2 (1.4%)	0	0	0	2 (1.4%)
Back pain	1 (0.7%)	0	0	0	1 (0.7%)
Vertigo	1 (0.7%)	0	0	0	1 (0.7%)
Peripheral vascular disease	1 (0.7%)	0	0	0	1 (0.7%)
Atrial fibrillation	1	0	0	0	1 (0.7%)
Mildly dilated LV	1	0	0	0	1 (0.7%)
Pancreatitis	1	0	0	0	1 (0.7%)
Nausea	1	0	0	0	1 (0.7%)
Pericardial effusion	1	0	0	0	1 (0.7%)
Leg edema	1	0	0	0	1 (0.7%)
Hirsutism	1	0	0	0	1 (0.7%)
Dermatitis	1	0	0	0	1 (0.7%)
Cellulitis	1	0	0	0	1 (0.7%)
Orthopnea	1	0	0	0	1 (0.7%)
Oral thrush	1	0	0	0	1 (0.7%)
Nephrolithiasis	1	0	0	0	1 (0.7%)
Hyponatremia	1	0	0	0	1 (0.7%)
Anxiety	1	0	0	0	1 (0.7%)
Pruritus	1	0	0	0	1 (0.7%)
Cholelithiasis	1	0	0	0	1 (0.7%)
Angina	1	0	0	0	1 (0.7%)
Digital desquamation	1	0	0	0	1 (0.7%)
Sinusitis	1	0	0	0	1 (0.7%)
Constipation	1	0	0	0	1 (0.7%)
Leg/hand pain	1	0	0	0	1 (0.7%)
Scalp pain	1	0	0	0	1 (0.7%)
Oral wounds	1	0	0	0	1 (0.7%)
Deep vein thrombosis	1	0	0	0	1 (0.7%)
Itchy scalp	1	0	0	0	1 (0.7%)
Mouth sores	1	0	0	0	1 (0.7%)
Stomatitis	1	0	0	0	1 (0.7%)

Subgroup analysis based on VAF demonstrated a similar predominance of low-grade AEs. In patients with VAF >30%, fatigue was the most common AE (29.6%), primarily grade 1–2. Diarrhea (21.3%) and rash (18.0%) were also frequent, without any grade 3–4 events. Dyspnea (16.4%) and cough (8.2%) were reported exclusively as grade 1. Other AEs, including decreased appetite, paronychia, pancytopenia, pain symptoms, nausea, and neutropenia, occurred less frequently (3.3%–4.9%) and were mostly low grade. Rare events (≤1.6%) included pneumonitis, pericardial effusion, atrial fibrillation, dermatologic, metabolic, and musculoskeletal toxicities. Notably, no grade 3–4 events were observed for most AEs, supporting an overall favorable tolerability profile (Table 3).

**TABLE 3:** Treatment-related adverse events of patients with VAF ≥30.

Adverse event	Grade 1 (frequency, %)	Grade 2 (frequency, %)	Grade 3 and 4 (frequency, %)	Total (frequency, %)
Fatigue	14 (23.0%)	4 (6.6%)	0 (0%)	18 (29.6%)
Diarrhea	8 (13.1%)	5 (8.2%)	0 (0%)	13 (21.3%)
Rash	6 (9.8%)	5 (8.2%)	0 (0%)	11 (18.0%)
Dyspnea	5 (8.2%)	0 (0%)	0 (0%)	10 (16.4%)
Cough	5 (8.2%)	0 (0%)	0 (0%)	5 (8.2%)
Decreasing heart ejection fraction	3 (4.9%)	0 (0%)	0 (0%)	3 (4.9%)
Decreased appetite	3 (4.9%)	0 (0%)	0 (0%)	3 (4.9%)
Paronychia	3 (4.9%)	0 (0%)	0 (0%)	3 (4.9%)
Pancytopenia	2 (3.3%)	1 (1.6%)	0 (0%)	3 (4.9%)
Back pain	2 (3.3%)	0 (0%)	0 (0%)	2 (3.3%)
Abdominal pain	2 (3.3%)	0 (0%)	0 (0%)	2 (3.3%)
Nausea	2 (3.3%)	0 (0%)	0 (0%)	2 (3.3%)
Neutropenia	2 (3.3%)	0 (0%)	0 (0%)	2 (3.3%)
Hair loss	0 (0%)	2 (3.3%)	0 (0%)	2 (3.3%)
Thrombocytopenia	0 (0%)	2 (3.3%)	0 (0%)	2 (3.3%)
Anemia	1 (1.6%)	1 (1.6%)	0 (0%)	2 (3.3%)
Pneumonitis	1 (1.6%)	0 (0%)	0 (0%)	1 (1.6%)
Leg/hand pain	1 (1.6%)	0 (0%)	0 (0%)	1 (1.6%)
Chest pain	1 (1.6%)	0 (0%)	0 (0%)	1 (1.6%)
Pericardial effusion	1 (1.6%)	0 (0%)	0 (0%)	1 (1.6%)
Atrial fibrillation	1 (1.6%)	0 (0%)	0 (0%)	1 (1.6%)
Leg edema	1 (1.6%)	0 (0%)	0 (0%)	1 (1.6%)
Vomiting	1 (1.6%)	0 (0%)	0 (0%)	1 (1.6%)
Dermatitis	1 (1.6%)	0 (0%)	0 (0%)	1 (1.6%)
Cellulitis	1 (1.6%)	0 (0%)	0 (0%)	1 (1.6%)
Orthopnea	1 (1.6%)	0 (0%)	0 (0%)	1 (1.6%)
Pruritus	1 (1.6%)	0 (0%)	0 (0%)	1 (1.6%)
Anxiety	1 (1.6%)	0 (0%)	0 (0%)	1 (1.6%)
Pancreatitis	1 (1.6%)	0 (0%)	0 (0%)	1 (1.6%)
Arthralgia	1 (1.6%)	0 (0%)	0 (0%)	1 (1.6%)
Mild dyslipidemia	1 (1.6%)	0 (0%)	0 (0%)	1 (1.6%)
Hirsutism	1 (1.6%)	0 (0%)	0 (0%)	1 (1.6%)
Sinusitis	1 (1.6%)	0 (0%)	0 (0%)	1 (1.6%)
Transaminases elevated	1 (1.6%)	0 (0%)	0 (0%)	1 (1.6%)
Elevated creatinine	1 (1.6%)	0 (0%)	0 (0%)	1 (1.6%)
Hypercalcemia	1 (1.6%)	0 (0%)	0 (0%)	1 (1.6%)
Headache	0 (0%)	1 (1.6%)	0 (0%)	1 (1.6%)
Peripheral neuropathy	0 (0%)	1 (1.6%)	0 (0%)	1 (1.6%)
Stomatitis	0 (0%)	1 (1.6%)	0 (0%)	1 (1.6%)
Fatigue	14 (23.0%)	4 (6.6%)	0 (0%)	18 (29.6%)
Diarrhea	8 (13.1%)	5 (8.2%)	0 (0%)	13 (21.3%)
Rash	6 (9.8%)	5 (8.2%)	0 (0%)	11 (18.0%)
Dyspnea	5 (8.2%)	0 (0%)	0 (0%)	10 (16.4%)
Cough	5 (8.2%)	0 (0%)	0 (0%)	5 (8.2%)
Decreasing heart ejection fraction	3 (4.9%)	0 (0%)	0 (0%)	3 (4.9%)
Decreased appetite	3 (4.9%)	0 (0%)	0 (0%)	3 (4.9%)
Paronychia	3 (4.9%)	0 (0%)	0 (0%)	3 (4.9%)

Among patients with VAF <30%, AEs were likewise predominantly grade 1–2. The most common events included fatigue (20.7%), rash (12.6%), anemia (9.2%), and paronychia (9.2%), with occasional grade 3 cases. Cough (8.0%), diarrhea (5.7%), and mild renal impairment (5.7%) were also reported. Events occurring in approximately 3.4% of patients included creatinine elevation, dyspnea, chest pain, abdominal pain, oral mucosal lesions, and thrombocytopenia, the latter associated with single grade 4 case. A broad spectrum of less frequent AEs (1.1%) was observed, almost exclusively grade 1. Rare but clinically relevant events included grade 4 pneumonitis and grade 3 neutropenia/leukopenia (Table 4).

**TABLE 4:** Treatment-related adverse events of patients with VAF <30.

Adverse event	Grade 1 (frequency, %)	Grade 2 (frequency, %)	Grade 3 (frequency, %)	Grade 4 (frequency, %)	Total (%)
Fatigue	13 (14.9%)	4 (4.6%)	1 (1.1%)	0 (0%)	18 (20.7%)
Rash	6 (6.9%)	4 (4.6%)	1 (1.1%)	0 (0%)	11 (12.6%)
Mild anemia	4 (4.6%)	3 (3.4%)	1 (1.1%)	0 (0%)	8 (9.2%)
Paronychia	3 (3.4%)	4 (4.6%)	1 (1.1%)	0 (0%)	8 (9.2%)
Cough	7 (8.0%)	0 (0%)	0 (0%)	0 (0%)	7 (8.0%)
Diarrhea	3 (3.4%)	1 (1.1%)	1 (1.1%)	0 (0%)	5 (5.7%)
Mild RF	5 (5.7%)	0 (0%)	0 (0%)	0 (0%)	5 (5.7%)
Creatinine elevation	4 (4.6%)	0 (0%)	0 (0%)	0 (0%)	4 (4.6%)
Dyspnea	3 (3.4%)	0 (0%)	0 (0%)	0 (0%)	3 (3.4%)
Chest pain	3 (3.4%)	0 (0%)	0 (0%)	0 (0%)	3 (3.4%)
Abdominal pain	3 (3.4%)	0 (0%)	0 (0%)	0 (0%)	3 (3.4%)
Oral wounds/sores	2 (2.3%)	1 (1.1%)	0 (0%)	0 (0%)	3 (3.4%)
Thrombocytopenia	2 (2.3%)	0 (0%)	0 (0%)	1 (1.1%)	3 (3.4%)
Pruritus	2 (2.3%)	0 (0%)	0 (0%)	0 (0%)	2 (2.3%)
Vertigo	2 (2.3%)	0 (0%)	0 (0%)	0 (0%)	2 (2.3%)
Hyponatremia	1 (1.1%)	0 (0%)	0 (0%)	0 (0%)	1 (1.1%)
Hypercalcemia	1 (1.1%)	0 (0%)	0 (0%)	0 (0%)	1 (1.1%)
Hearing decline	1 (1.1%)	0 (0%)	0 (0%)	0 (0%)	1 (1.1%)
Digital desquamation	1 (1.1%)	0 (0%)	0 (0%)	0 (0%)	1 (1.1%)
Itchy scalp	1 (1.1%)	0 (0%)	0 (0%)	0 (0%)	1 (1.1%)
Scalp pain	1 (1.1%)	0 (0%)	0 (0%)	0 (0%)	1 (1.1%)
Alopecia	1 (1.1%)	0 (0%)	0 (0%)	0 (0%)	1 (1.1%)
DVT	1 (1.1%)	0 (0%)	0 (0%)	0 (0%)	1 (1.1%)
Constipation	1 (1.1%)	0 (0%)	0 (0%)	0 (0%)	1 (1.1%)
Angina	1 (1.1%)	0 (0%)	0 (0%)	0 (0%)	1 (1.1%)
Decreasing heart ejection fraction	1 (1.1%)	0 (0%)	0 (0%)	0 (0%)	1 (1.1%)
PVD	1 (1.1%)	0 (0%)	0 (0%)	0 (0%)	1 (1.1%)
Headache	1 (1.1%)	0 (0%)	0 (0%)	0 (0%)	1 (1.1%)
Nausea	1 (1.1%)	0 (0%)	0 (0%)	0 (0%)	1 (1.1%)
Decreased appetite	1 (1.1%)	0 (0%)	0 (0%)	0 (0%)	1 (1.1%)
Arthralgia	1 (1.1%)	0 (0%)	0 (0%)	0 (0%)	1 (1.1%)
Pancytopenia	1 (1.1%)	0 (0%)	0 (0%)	0 (0%)	1 (1.1%)
Dyslipidemia	1 (1.1%)	0 (0%)	0 (0%)	0 (0%)	1 (1.1%)
Nephrolithiasis	1 (1.1%)	0 (0%)	0 (0%)	0 (0%)	1 (1.1%)
Pneumonitis	0 (0%)	0 (0%)	0 (0%)	1 (1.1%)	1 (1.1%)
Neutropenia/Leukopenia	0 (0%)	0 (0%)	1 (1.1%)	0 (0%)	1 (1.1%)

Comparative analysis between VAF subgroups showed largely similar AE profiles. However, diarrhea (21.3% vs. 5.7%, p = 0.005) and dyspnea (16.4% vs. 3.4%, p = 0.0085) were significantly more common in the high VAF group. Conversely, anemia (9.2% vs. 3.3%, p = 0.03) and creatinine elevation (5.7% vs. 1.6%, p = 0.01) were more frequent in the low VAF group. No other statistically significant differences were identified. Overall, these findings suggest that the safety profile of osimertinib remains consistent regardless of VAF status (Table 5).

**TABLE 5:** Compares treatment-related adverse events between patients with VAF <30% and VAF ≥30%.

Adverse event	Patients with VAF≥30, (61, 41.5%)	Patients with VAF<30, (86, 58.5%)	P value
Fatigue	18 (29.6%)	18 (20.7%)	0.2487
Diarrhea	13 (21.3%)	5 (5.7%)	0.005
Rash	11 (18.0%)	11 (12.6%)	0.4824
Dyspnea	10 (16.4%)	3 (3.4%)	0.0085
Cough	5 (8.2%)	7 (8.0%)	1
Paronychia	3 (4.9%)	8 (9.2%)	0.3629
Decreased appetite	3 (4.9%)	—	0.0694
Decreased EF	3 (4.9%)	—	0.0694
Pancytopenia	3 (4.9%)	—	0.0694
Back pain	2 (3.3%)	—	0.1705
Abdominal pain	2 (3.3%)	3 (3.4%)	1
Nausea	2 (3.3%)	1 (1.1%)	0.5701
Neutropenia	2 (3.3%)	1 (1.1%)	0.5701
Hair loss	2 (3.3%)	—	0.1705
Thrombocytopenia	2 (3.3%)	1 (1.1%)	0.5701
Anemia	2 (3.3%)	8 (9.2%)	0.03
Pneumonitis	1 (1.6%)	1 (1.1%)	1
Leg/hand pain	1 (1.6%)	—	0.4149
Chest pain	1 (1.6%)	3 (3.4%)	0.6474
Pericardial effusion	1 (1.6%)	—	0.4149
Atrial fibrillation	1 (1.6%)	1 (1.1%)	1
Leg edema	1 (1.6%)	—	0.4149
Vomiting	1 (1.6%)	2 (2.3%)	1
Dermatitis	1 (1.6%)	4 (4.6%)	0.403
Cellulitis	1 (1.6%)	—	0.415
Orthopnea	1 (1.6%)	1 (1.1%)	1
Pruritus	1 (1.6%)	2 (2.3%)	1
Anxiety	1 (1.6%)	—	0.415
Pancreatitis	1 (1.6%)	—	0.415
Arthralgia	1 (1.6%)	2 (2.3%)	1
Mild dyslipidemia	1 (1.6%)	2 (2.3%)	1
Hirsutism	1 (1.6%)	1 (1.1%)	1
Sinusitis	1 (1.6%)	1 (1.1%)	1
Transaminases elevation	1 (1.6%)	—	0.415
Creatinine elevation	1 (1.6%)	5 (5.7%)	0.01
Hypercalcemia	1 (1.6%)	1 (1.1%)	1
Headache	1 (1.6%)	1 (1.1%)	1
Peripheral neuropathy	1 (1.6%)	1 (1.1%)	1
Stomatitis	1 (1.6%)	3 (3.4%)	0.6418

## 4 Discussion

Lung cancer remains one of the leading causes of cancer-related mortality worldwide, with NSCLC accounting for the majority of cases. Despite advances in screening and treatment, many patients are still diagnosed at advanced stages, underscoring the need for continuous optimization of therapeutic strategies. Over the past decade, the paradigm shift toward precision oncology has fundamentally transformed NSCLC management, particularly through the identification of actionable molecular alterations.

In this context, precise tumor characterization—integrating histologic subtyping with comprehensive molecular profiling—has become a cornerstone of clinical decision-making. Among the most clinically relevant targets is EGFR, particularly in lung adenocarcinoma. Multiple studies have demonstrated substantial benefit from EGFR tyrosine kinase inhibitors (TKIs), especially osimertinib, in patients with advanced or locally advanced NSCLC harboring sensitizing EGFR mutations. However, clinical outcomes remain heterogeneous, with patients carrying similar EGFR alterations experiencing markedly different progression-free survival (PFS) and overall survival (OS) (Zhang et al., 2016; Tan et al., 2015; Tan et al., 2018).

VAF provides a quantitative measure of the proportion of tumor cells carrying a given mutation and adds an important layer of biological information beyond mutation status alone. VAF is influenced by several factors, including tumor cellularity, copy number alterations, and the proportion of tumor cells harboring the mutation. Early truncal or clonal mutations are typically present in a large fraction of tumor cells and therefore exhibit higher VAF, whereas later subclonal events tend to occur in only a subset of cells and demonstrate lower frequencies. Consequently, a high EGFR VAF generally indicates that the mutation is a dominant clonal driver present in the majority of cancer cells, reflecting a more homogeneous tumor architecture and greater dependence on EGFR-mediated oncogenic signaling. In contrast, tumor heterogeneity refers to the coexistence of genetically distinct subclonal populations within the same tumor, some of which may not rely on EGFR signaling or may harbor intrinsic resistance mechanisms. Therefore, while high VAF is often associated with lower functional heterogeneity and a more uniform therapeutic target, low VAF may serve as an indirect marker of increased subclonality and genomic complexity. Given that truncal mutations are more likely to drive tumor growth and survival, they may represent more effective targets for EGFR-directed therapies, whereas increased heterogeneity and lower VAF have been associated with inferior responses and earlier resistance to Osimertinib (Friedlaender et al., 2021; Gieszer et al., 2021; Ramalingam et al., 2020; Gerlinger et al., 2012).

Despite this biological rationale, the clinical relevance of EGFR VAF in NSCLC remains incompletely defined, particularly in the context of prognosis and response to osimertinib. Available data are limited and sometimes conflicting. Mechanistically, higher VAF may reflect greater tumor dependence on EGFR signaling, potentially translating into enhanced sensitivity to EGFR inhibition; however, this hypothesis has not been consistently validated in clinical studies.

Evidence from prior studies highlights this uncertainty. A retrospective analysis of the phase III CTONG 0901 trial, comparing erlotinib and gefitinib in patients with EGFR exon 19 deletions or exon 21 L858R mutations, reported a median allelic frequency of 25.8% (range, 1.4%–86.2%) in 105 patients. When stratified by VAF, no significant differences were observed in objective response rate (56.2% vs. 57.5%), PFS (11.2 vs. 12.4 months), or OS (20.5 vs. 23.1 months; P = 0.500) (Ramalingam et al., 2020). In contrast, a smaller study of 42 patients treated with various first-line EGFR TKIs suggested that higher allelic frequency (>0.30) was associated with improved PFS, although no OS benefit was observed (Friedlaender et al., 2021). Similarly, a real-world cohort of 89 patients demonstrated a positive correlation between EGFR VAF and PFS (r = 0.319; P = 0.002), with high-VAF patients (≥70%) experiencing significantly longer PFS (52 vs. 26 weeks; P < 0.001) and improved OS (P = 0.011), although most patients received first- or second-generation TKIs (Gieszer et al., 2021).

With the establishment of osimertinib as the preferred first-line standard of care following the FLAURA and FLAURA 2 trials (Gregorc et al., 2018; Paz-Ares et al., 2010; O'Sullivan et al., 2022), earlier evidence derived from prior-generation TKIs is less representative of current practice. Our study addresses this gap by focusing exclusively on patients treated with first-line osimertinib and represents, to our knowledge, the largest real-world analysis in this setting.

In our cohort, higher EGFR VAF was consistently associated with improved PFS and OS in both the overall population and key molecular subgroups, including exon 19 deletions and exon 21 L858R mutations. These findings support the potential role of VAF as a prognostic biomarker in patients treated with osimertinib. However, because all patients received the same agent, it remains uncertain whether VAF is predictive of osimertinib-specific benefit or instead reflects broader tumor biology, including clonality and intratumoral heterogeneity.

To assess the robustness of the selected VAF threshold, we performed an exploratory minimal p-value analysis across a range of candidate cut-off values. The threshold associated with the greatest separation in overall survival was approximately 33%, which closely aligned with the 30% cut-off used in our primary analyses. While VAF is inherently a continuous variable, defining a clinically meaningful threshold may facilitate risk stratification and improve clinical interpretability in routine clinical practice.

Biologically, the association between higher VAF and improved outcomes may be explained by tumor clonality. High VAF likely reflects dominant EGFR-driven clones and reduced intratumoral heterogeneity, both of which have been associated with enhanced response to targeted therapies. Supporting this concept, prior work, including that by Buder et al., has demonstrated improved outcomes in patients with higher EGFR mutation allele frequency, even in the post-TKI setting (Kunimasa et al., 2022). Single-cell analyses further suggest that tumors with a higher proportion of EGFR-mutant cells exhibit reduced heterogeneity and better therapeutic response, whereas increased heterogeneity is associated with resistance (Lin et al., 2023). Broader genomic analyses also support VAF as a surrogate of tumor clonality, where lower allele fractions may indicate subclonal mutations with reduced therapeutic dependency (Provencio et al., 2017).

Overall, toxicity profiles were broadly comparable between VAF subgroups, with predominantly low-grade (grade 1–2) adverse events observed in both groups, consistent with the established safety profile of osimertinib reported in pivotal trials (Ramalingam et al., 2020; Gerlinger et al., 2012; Gregorc et al., 2018). No meaningful difference in severe (grade ≥3) toxicity was identified, suggesting that VAF does not significantly affect overall tolerability.

However, distinct patterns of specific adverse events emerged. Higher VAF (>30%) was associated with increased rates of diarrhea and dyspnea, whereas anemia and creatinine elevation were more frequent in the low VAF group (<30%). These findings may reflect differences in tumor biology and EGFR dependency. Higher VAF likely indicates stronger reliance on EGFR signaling, resulting in more effective pathway inhibition by osimertinib. On-target inhibition of EGFR in normal epithelial tissues—particularly within the gastrointestinal and respiratory tracts—may therefore explain increased diarrhea and dyspnea, consistent with the known role of EGFR in epithelial homeostasis and barrier integrity (Wang et al., 2025; Ariyadamrongkwan and Muanprasat, 2025; Agbarya et al., 2026).

Conversely, the higher incidence of anemia and renal dysfunction in low-VAF tumors may reflect greater tumor heterogeneity and alternative oncogenic drivers, which are often associated with more aggressive disease biology and systemic effects (Kunimasa et al., 2022; Lin et al., 2023). Reduced EGFR dependency may also translate into less effective tumor control, contributing to disease-related complications and laboratory abnormalities (Friedlaender et al., 2021; Gieszer et al., 2021).

Prior landmark studies, including FLAURA, have reported diarrhea, rash, and fatigue as the most common adverse events with osimertinib, generally low grade and without stratification by molecular features such as VAF (Ramalingam et al., 2020; Gerlinger et al., 2012; Gregorc et al., 2018; Paz-Ares et al., 2010). To our knowledge, no previous study has systematically evaluated the relationship between EGFR VAF and toxicity patterns, making our findings novel in suggesting that allelic burden may influence not only efficacy but also treatment-related toxicity profiles.

From a mechanistic perspective, EGFR signaling plays a fundamental role in epithelial proliferation and tissue repair across multiple organ systems. Its inhibition leads to disruption of epithelial integrity, increased permeability, and inflammatory changes. In the gastrointestinal tract, EGFR-TKI–induced diarrhea is mediated by altered ion transport, impaired barrier function, and microbiome changes, collectively resulting in fluid loss and mucosal injury (Ariyadamrongkwan and Muanprasat, 2025). Cutaneous toxicities, including rash and paronychia, result from impaired keratinocyte proliferation and follicular inflammation (Recuero et al., 2023). These toxicities are therefore considered on-target effects of EGFR inhibition.

More broadly, TKI-associated adverse events are increasingly understood as mechanism-based toxicities arising from pathway inhibition in normal tissues rather than nonspecific off-target effects (Shyam Sunder et al., 2023). In this framework, higher VAF tumors—being more EGFR-dependent—may exhibit more pronounced on-target toxicities due to more effective pathway suppression. In contrast, lower VAF tumors, characterized by greater heterogeneity and reduced EGFR dependence, may demonstrate attenuated epithelial toxicity but increased systemic or disease-related adverse effects.

Importantly, these findings remain reassuring from a clinical standpoint, as osimertinib demonstrated a favorable safety profile across all VAF strata. Nevertheless, the observed differences in toxicity patterns may have practical implications. Patients with higher VAF may benefit from closer monitoring for gastrointestinal and respiratory symptoms, whereas those with lower VAF may require more vigilant assessment of hematologic and renal parameters.

Patients with higher EGFR VAF are generally associated with longer survival and may consequently remain on osimertinib treatment for a longer duration. This extended treatment exposure could increase the likelihood of detecting or reporting adverse events, as well as the cumulative number of events observed during follow-up. Therefore, the observed differences in toxicity patterns may, at least in part, reflect differences in treatment duration and exposure time rather than a direct biological association between VAF and adverse event development.

Several limitations should be acknowledged. The retrospective design and lack of randomization introduce potential bias. In addition, no universally accepted VAF threshold exists. Our cut-off was derived from histopathologic tumor cellularity estimates, assuming that a fully clonal mutation approximates a 50% allele frequency. While biologically plausible, this assumption may be affected by copy number alterations and tumor purity, and its generalizability requires further validation.

Finally, our findings raise important clinical questions. It remains to be determined whether patients with low VAF (<30%) may benefit from combination strategies such as adding chemotherapy to osimertinib, and whether such approaches could further improve outcomes even in high-VAF tumors. Prospective studies are needed to clarify whether VAF can be integrated into treatment selection and to establish its role in guiding personalized therapeutic strategies in EGFR-mutant NSCLC. Also, several adverse event subgroup analyses were based on a small number of events, particularly for uncommon toxicities such as anemia and creatinine elevation. Therefore, statistically significant differences observed between VAF groups should be interpreted with caution, as they may reflect random variation arising from multiple comparisons and limited event counts. These findings should be considered exploratory and require confirmation in larger cohorts before any definitive association between VAF status and toxicity patterns can be established.

## 5 Conclusion

Our study evaluated the relationship between EGFR VAF and both efficacy and treatment-related adverse events in patients with EGFR-mutant NSCLC treated with first-line osimertinib. Higher VAF (≥30%) was significantly associated with improved overall survival and demonstrated a consistent numerical trend toward longer progression-free survival across the overall cohort and key molecular subgroups. Importantly, osimertinib maintained a favorable and largely comparable safety profile regardless of VAF status, with predominantly low-grade toxicities observed in both groups. However, distinct differences in specific adverse event patterns were identified, suggesting that VAF may influence the spectrum rather than the overall incidence of toxicity. These findings support the hypothesis that VAF reflects tumor clonality and biological dependence on EGFR signaling, which may contribute to differences in treatment outcomes and toxicity profiles. Overall, EGFR VAF may serve as a prognostic biomarker and a potential indicator of toxicity patterns, warranting further prospective validation before incorporation into routine clinical decision-making.

## Data Availability

The data presented in this study are available on request from the corresponding authors.

## References

[B1] Agbarya A. Mhameed K. Soklakova A. Nasrallah H. Abu Amna M. El-Saied S. (2026). Impact of variant allele frequency (VAF) levels on clinical efficacy of osimertinib in patients with metastatic NSCLC. Med. Sci. (Basel) 14 (2), 233. 10.3390/medsci14020233 42201025 PMC13214930

[B2] Ariyadamrongkwan J. Muanprasat C. (2025). Pathophysiological mechanisms underlying diarrhea across generations of EGFR-TKIs: the role of ERBB signaling and potential therapies. Biomed. Pharmacother. 192, 118562. Epub 2025 Sep 18. 10.1016/j.biopha.2025.118562 40972401

[B3] Cross D. A. Ashton S. E. Ghiorghiu S. Eberlein C. Nebhan C. A. Spitzler P. J. (2014). AZD9291, an irreversible EGFR TKI, overcomes T790M-mediated resistance to EGFR inhibitors in lung cancer. Cancer Discov. 4 (9), 1046–1061. Epub 2014 Jun 3. 10.1158/2159-8290.CD-14-0337 24893891 PMC4315625

[B4] D'Angelo S. P. Pietanza M. C. Johnson M. L. Riely G. J. Miller V. A. Sima C. S. (2011). Incidence of EGFR exon 19 deletions and L858R in tumor specimens from men and cigarette smokers with lung adenocarcinomas. J. Clin. Oncol. 29 (15), 2066–2070. Epub 2011 Apr 11. 10.1200/JCO.2010.32.6181 21482987 PMC3296671

[B5] Friedlaender A. Tsantoulis P. Chevallier M. De Vito C. Addeo A. (2021). The impact of variant allele frequency in EGFR mutated NSCLC patients on targeted therapy. Front. Oncol. 11, 644472. 10.3389/fonc.2021.644472 33869038 PMC8044828

[B6] Gerlinger M. Rowan A. J. Horswell S. Math M. Larkin J. Endesfelder D. (2012). Intratumor heterogeneity and branched evolution revealed by multiregion sequencing. N. Engl. J. Med. 366 (10), 883–892. 10.1056/NEJMoa1113205 22397650 PMC4878653

[B7] Gieszer B. Megyesfalvi Z. Dulai V. Papay J. Kovalszky I. Timar J. (2021). EGFR variant allele frequency predicts EGFR-TKI efficacy in lung adenocarcinoma: a multicenter study. Transl. Lung Cancer Res. 10 (2), 662–674. 10.21037/tlcr-20-814 33718012 PMC7947383

[B8] Gregorc V. Lazzari C. Karachaliou N. Rosell R. Santarpia M. (2018). Osimertinib in untreated epidermal growth factor receptor (*EGFR*)-Mutated advanced non-small cell lung cancer. Transl. Lung Cancer Res. 7 (Suppl. 2), S165–S170. 10.21037/tlcr.2018.03.19 29782558 PMC5943214

[B9] Kunimasa K. Nishino K. Sato Y. Mori M. Ihara S. Suzuki H. (2022). Fragment size and dynamics of EGFR-Mutated tumor-derived DNA provide prognostic information regarding EGFR-TKI efficacy in patients with EGFR-Mutated NSCLC. Sci. Rep. 12 (1), 13544. 10.1038/s41598-022-17848-y 35941190 PMC9360008

[B10] Lamb Y. N. (2021). Osimertinib: a review in previously untreated, EGFR mutation-positive, advanced NSCLC. Target Oncol. 16 (5), 687–695. 10.1007/s11523-021-00839-w 34564820 PMC8536603

[B11] Lin Z. Li Y. Tang S. Deng Q. Jiang J. Zhou C. (2023). Comparative analysis of genomic profiles between tissue-based and plasma-based next-generation sequencing in patients with non-small cell lung cancer. Lung Cancer 182, 107282. Epub 2023 Jun 22. 10.1016/j.lungcan.2023.107282 37392713

[B12] Meng Y. Bai R. Cui J. (2023). Precision targeted therapy for EGFR mutation-positive NSCLC: dilemmas and coping strategies. Thorac. Cancer 14 (13), 1121–1134. Epub 2023 Apr 2. 10.1111/1759-7714.14858 37005552 PMC10151140

[B13] National Comprehensive Cancer Network, Clinical Practice Guidelines in Oncology, Non-small Cell Lung Cancer (2025). Available online at: https://crain-platform-precisiononcologynews-prod.s3.amazonaws.com/2024-04/nscl_NCCN%20guidelines.pdf (Accessed on 5 May 2024).

[B14] O'Sullivan D. E. Jarada T. N. Yusuf A. Hu L. X. Y. Gogna P. Brenner D. R. (2022). Prevalence, treatment patterns, and outcomes of individuals with *EGFR* positive metastatic non-small cell lung cancer in a Canadian real-world setting: a comparison of exon 19 deletion, L858R, and exon 20 insertion *EGFR* mutation carriers. Curr. Oncol. 29 (10), 7198–7208. 10.3390/curroncol29100567 36290844 PMC9600059

[B15] Paz-Ares L. Soulières D. Melezínek I. Moecks J. Keil L. Mok T. (2010). Clinical outcomes in non-small-cell lung cancer patients with EGFR mutations: pooled analysis. J. Cell Mol. Med. 14 (1-2), 51–69. Epub 2009 Dec 8. 10.1111/j.1582-4934.2009.00991.x 20015198 PMC3837609

[B16] Provencio M. Torrente M. Calvo V. Pérez-Callejo D. Gutiérrez L. Franco F. (2017). Prognostic value of quantitative ctDNA levels in non small cell lung cancer patients. Oncotarget 9 (1), 488–494. 10.18632/oncotarget.22470 29416630 PMC5787483

[B17] Ramalingam S. S. Vansteenkiste J. Planchard D. Cho B. C. Gray J. E. Ohe Y. (2020). Overall survival with osimertinib in untreated, *EGFR*-mutated advanced NSCLC. N. Engl. J. Med. 382 (1), 41–50. Epub 2019 Nov 21. 10.1056/NEJMoa1913662 31751012

[B18] Recuero J. K. Fitz J. R. Pereira A. A. Bonamigo R. R. (2023). EGFR inhibitors: clinical aspects, risk factors and biomarkers for acneiform eruptions and other mucosal and cutaneous adverse effects. Bras Dermatol 98 (4), 429–439. Epub 2023 Mar 27. 10.1016/j.abd.2022.10.004 36990917 PMC10334360

[B19] Shalata W. Maimon Rabinovich N. Agbarya A. Yakobson A. Dudnik Y. Abu J. A. (2024). Efficacy of pembrolizumab vs. nivolumab plus ipilimumab in metastatic NSCLC in relation to PD-L1 and TMB status. Cancers (Basel) 16 (10), 1825. 10.3390/cancers16101825 38791905 PMC11119071

[B20] Shalata W. Iraqi M. Bhattacharya B. Fuchs V. Roisman L. C. Cohen A. Y. (2021). Rapid response to the combination of lenvatinib and pembrolizumab in patients with advanced carcinomas (lung adenocarcinoma and malignant pleural mesothelioma). Cancers (Basel) 13 (14), 3630. 10.3390/cancers13143630 34298855 PMC8307809

[B21] Shalata W. Krayim B. Miari A. Agbarya A. Peled N. Dudnik Y. (2026). Variant allele frequency as a predictor of treatment response to osimertinib in EGFR-Mutated NSCLC. Front. Mol. Biosci. 13, 1822285. 10.3389/fmolb.2026.1822285 42109522 PMC13152828

[B22] Shyam Sunder S. Sharma U. C. Pokharel S. (2023). Adverse effects of tyrosine kinase inhibitors in cancer therapy: pathophysiology, mechanisms and clinical management. Signal Transduct. Target Ther. 8 (1), 262. 10.1038/s41392-023-01469-6 37414756 PMC10326056

[B23] Soria J. C. Ohe Y. Vansteenkiste J. Reungwetwattana T. Chewaskulyong B. Lee K. H. (2018). Osimertinib in untreated EGFR-mutated advanced non-small-cell lung cancer. N. Engl. J. Med. 378 (2), 113–125. Epub 2017 Nov 18. 10.1056/NEJMoa1713137 29151359

[B24] Sung H. Ferlay J. Siegel R. L. Laversanne M. Soerjomataram I. Jemal A. (2021). Global cancer statistics 2020: GLOBOCAN estimates of incidence and mortality worldwide for 36 cancers in 185 countries. CA Cancer J. Clin. 71 (3), 209–249. Epub 2021 Feb 4. 10.3322/caac.21660 33538338

[B25] Tan C. S. Gilligan D. Pacey S. (2015). Treatment approaches for EGFR-inhibitor-resistant patients with non-small-cell lung cancer. Lancet Oncol. 16 (9), e447–e459. 10.1016/S1470-2045(15)00246-6 26370354

[B26] Tan C. S. Kumarakulasinghe N. B. Huang Y. Q. Ang Y. L. E. Choo J. R. Goh B. C. (2018). Third generation EGFR TKIs: current data and future directions. Mol. Cancer 17 (1), 29. 10.1186/s12943-018-0778-0 29455654 PMC5817792

[B27] Wang R. Chen Y. Li L. Zhang L. Zhang S. (2025). Osimertinib acquired resistance among patients with EGFR-Mutated NSCLC: from molecular mechanisms to clinical therapeutic strategies. Cancer Drug Resist. 8, 61. 10.20517/cdr.2025.140 41425254 PMC12713175

[B28] Zhang Y. L. Yuan J. Q. Wang K. F. Fu X. H. Han X. R. Threapleton D. (2016). The prevalence of EGFR mutation in patients with non-small cell lung cancer: a systematic review and meta-analysis. Oncotarget 7 (48), 78985–78993. 10.18632/oncotarget.12587 27738317 PMC5346692

